# Supplementary material for: Content-rich biological network constructed by mining PubMed abstracts
Source: BMC Bioinformatics. 2004 Oct 8;5:147. doi: 10.1186/1471-2105-5-147 (PMC528731; doi:10.1186/1471-2105-5-147)
Supplement: Additional File 2 — The original results of the above study (non-essential files are deleted to keep the file size under the limit set by BMC bioinformatics). [file 1471-2105-5-147-S2.bz2 › chilibotAdditionalFile2/dip05/604ID9729040E10983/html/H2_B2M.html]

 


 **H2** and **B2M** 
  
Found 20 abstracts in PubMed, retrieved 05.  
 

 What does Google say? 
 PDF only 
| .edu only 

---

**Interactive relationship** (e.g. stimulation, inhibition, etc)

**Neutral relationship**- Expression of each HLA hyb molecule and HLA B27 nat human beta 2 microglobulin  [ **B2M** ]  led to peripheral CD8 T cell levels comparable with that for mice expressing a single  **H2**  K b or  **H2**  D b gene.  Ref: 12421934 J Immunol, 2002

**Non-interactive relationship** (e.g. studied together, co-existance, homology, etc.)

- We previously described Tg mice carrying the HLA B27, HLA B7, or HLA A2 alleles expressed as fully native HLA nat with human beta 2 microglobulin  [ **B2M** ]  and as hybrid human mouse HLA hyb molecules on the  **H2**  b background.  Ref: 12421934 J Immunol, 2002
